# Twelve newly assembled jasmine chloroplast genomes: unveiling genomic diversity, phylogenetic relationships and evolutionary patterns among Oleaceae and *Jasminum* species

**DOI:** 10.1186/s12870-024-04995-9

**Published:** 2024-04-25

**Authors:** Xiuming Xu, Hechen Huang, Shaoqing Lin, Linwei Zhou, Yuchong Yi, Enwen Lin, Liqing Feng, Yu Zheng, Aiting Lin, Liying Yu, Yingjia Shen, Robert J. Henry, Jingping Fang

**Affiliations:** 1 College of Life Science, Fujian Normal University, Fuzhou, 350117 China; 2 Key Laboratory of the Ministry of Education for Coastal and Wetland Ecosystems, College of the Environment and Ecology, Xiamen University, Xiamen, 361102 China; 3 Queensland Alliance for Agriculture and Food Innovation, University of Queensland, Brisbane, Australia; 4 State Key Laboratory of Marine Environmental Science and College of Ocean and Earth Sciences, Xiamen University, Xiamen, 361102 China

**Keywords:** *Jasminum*, Jasmine, Chloroplast genome, Comparative analysis, Phylogenetic tree

## Abstract

**Background:**

Jasmine (*Jasminum*), renowned for its ornamental value and captivating fragrance, has given rise to numerous species and accessions. However, limited knowledge exists regarding the evolutionary relationships among various *Jasminum* species.

**Results:**

In the present study, we sequenced seven distinct *Jasminum* species, resulting in the assembly of twelve high-quality complete chloroplast (cp) genomes. Our findings revealed that the size of the 12 cp genomes ranged from 159 to 165 kb and encoded 134–135 genes, including 86–88 protein-coding genes, 38–40 tRNA genes, and 8 rRNA genes. *J. nudiflorum* exhibited a larger genome size compared to other species, mainly attributed to the elevated number of forward repeats (FRs). Despite the typically conservative nature of chloroplasts, variations in the presence or absence of *acc*D have been observed within *J. sambac*. The calculation of nucleotide diversity (*Pi*) values for 19 cp genomes indicated that potential mutation hotspots were more likely to be located in LSC regions than in other regions, particularly in genes *ycf*2, *rbc*L, *atp*E, *ndh*K, and *ndh*C (*Pi* > 0.2). Ka/Ks values revealed strong selection pressure on the genes *rps*2, *atp*A, *rpo*A, *rpo*C1, and *rpl*33 when comparing *J. sambac* with the three most closely related species (*J. auriculatum*, *J. multiflorum*, and *J. dichotomum*). Additionally, SNP identification, along with the results of Structure, PCA, and phylogenetic tree analyses, divided the *Jasminum* cp genomes into six groups. Notably, *J. polyanthum* showed gene flow signals from both the G5 group (*J. nudiflorum*) and the G3 group (*J. tortuosum* and *J. fluminense*). Phylogenetic tree analysis reflected that most species from the same genus clustered together with robust support in Oleaceae, strongly supporting the monophyletic nature of cp genomes within the genus *Jasminum*.

**Conclusion:**

Overall, this study provides comprehensive insights into the genomic composition, variation, and phylogenetic relationships among various *Jasminum* species. These findings enhance our understanding of the genetic diversity and evolutionary history of *Jasminum*.

**Supplementary Information:**

The online version contains supplementary material available at 10.1186/s12870-024-04995-9.

## Background

Oleaceae constitutes a nearly cosmopolitan family of trees, along with upright or climbing shrubs, which are classified under the Oleineae suborder within the subclass Metachlamydeae. This family includes over 400 species in 28 genera, being widely distributed in temperate and tropical regions. China showcases a diverse array of Oleaceae plants, with over 160 species across 10 genera [1]. As the largest genus within the Oleaceae family (about 200 species) [2], *Jasminum* possesses a wide range of characteristics, applications, and advantages, making it extensively cultivated for commercial purposes in many Asian countries. Additionally, they are commonly incorporated into bouquets and decorations [3, 4]. Among these species, *Jasminum sambac* stands out as a prized cultivated species, renowned for its ornamental, medicinal, and edible properties [3]. For over 1,500 years, *J. sambac* has been cultivated in China for its use in traditional Chinese medicine and the production of the famous “jasmine tea”. Its essential oil is extracted for use in the perfume industry and for the production of attars and hair oils [5, 6]. *J. sambac* plants typically exhibit three distinct phenotypes: single-petal (SP), double-petal (DP), and multi-petal (MP) [7]. Currently, DP varieties are commercially cultivated in various regions across China, including Fujian, Guangxi, Sichuan, Yunnan, Hainan, and Taiwan [8]. Despite the ornamental, ecological, and economic importance of *Jasminum* species, until recently little was known about the molecular diversity among them. Acquiring this information will contribute to the future breeding and conservation of jasmine.

The chloroplast (cp) is an organelle responsible for photosynthesis in plants. It contains electron carriers in its thylakoid membranes and all necessary enzymes in its stroma. It is hypothesized that chloroplasts evolved from cyanobacteria through endosymbiosis. Chloroplasts are also involved in the synthesis of amino acids, fatty acids, pigments, carbohydrates, and precursors for various hormones [9]. The cp genome (cpDNA) possesses a set of distinct properties, including its compact haploid size, abundant copy number, relatively stable gene number and organization, the absence of recombination, and maternal transmission [10, 11]. In angiosperms, most cp genomes are maternally inherited, while only a small number are inherited biparentally or paternally [12]. The cp genome has found extensive application in species phylogenetic classification and divergence time, owing to its high conservation [13]. Due to its small genome size and relatively conserved structure, cpDNA has become an ideal model for evolutionary and comparative genomic studies [14], providing more favorable evidence for uncovering the systematic position and genetic developmental relationships among various plant groups. With the development of next-generation DNA sequencing technologies, the complete cp genome has been widely used for plant identification, phylogenic analysis, and evolutionary studies.

Efforts have been dedicated to resolving the relationships among Oleaceae species. A comparative analysis of cp genome structures among various Oleaceae plants has also been undertaken [15]. In *Jasminum*, an evolutionary analysis using chloroplast markers have been carried out for 22 Indian jasmine species, revealing the monophyly of *Jasminum* when excluding *Menodora* spp. [2]. A total of 86 Olive cp genomes have been assembled, indicating incomplete lineage sorting and/or hybridization during the diversification of this extensive phylogenetic group, but only two *Jasminum* species were included in this analysis [16]. In addition, whole cp genome dataset of SNPs was employed to demonstrate that the tribe Oleeae originated via ancient hybridization and polyploidy [17]. *Jasminum* has morphologically been divided into four groups (*Alternifolia*, *Unifoliolata*, *Pinnatifolia*, and *Trifoliolata*) based on leaf arrangement and the number of leaflets [18]. However, recent systematic studies have identified five taxa and introduced additional *Primulina* sections, reclassifying some species previously grouped under *Pinnatifolia* as *Primulina* [19, 20].

Although previous researches have investigated the structural characteristics of the chloroplast genomes and phylogenetic relationships among Oleaceae plants, there still remains a dearth of cp genome data regarding *Jasminum* species. Within the genus *Jasminum*, complete cp genomes have been sequenced for only six species, including *Jasminum sambac*, *Jasminum fluminense*, *Jasminum fruticans*, *Jasminum nudiflorum*, *Jasminum tortuosum* and *Jasminum polyanthum*. Therefore, there is a need for additional species information to accurately ascertain the evolutionary relationships within the *Jasminum* genus and the Oleaceae family, as some nodes within the phylogeny are yet not fully resolved. A more comprehensive understanding of the differences in cp genome structure characteristics among *Jasminum* species will offer valuable perspectives on genomic diversity and future research on jasmine breeding.

In this study, we collected 12 *Jasminum* samples from seven significant germplasm resources of jasmine species, including *J. sambac*, *J. nudiflorum*, *Jasminum auriculatum*, *Jasminum dichotomum*, *Jasminum floridum*, *Jasminum multiflorum* and *Jasminum odoratissimum*. High-depth sequencing was performed on each sample, resulting in the assembly of 12 high-quality complete cp genomes. In addition, all publicly available complete cp genomes of *Jasminum* were retrieved, amassing a total of 19 samples representing 11 *Jasminum* species. We conducted intra- and inter-specific comparisons of these 19 complete cp genomes of *Jasminum* utilizing bioinformatics methods, including the analysis of genome structure and composition, genetic diversity, codon usage bias, long repeats, simple sequence repeats (SSRs), gene selection pressure, single nucleotide polymorphisms (SNPs) identification, and phylogenetic relationships within *Jasminum* and among all Oleaceae species. These analyses provide valuable insights into the distinct differences in chloroplast genome composition and variation among *Jasminum* species, as well as the evolutionary relationships and divergence among different Oleaceae and *Jasminum* species. This information can serve as an essential genomic foundation for breeding efforts in *Jasminum*.

## Materials and methods

### Sampling sites and sample collection

Plant materials from diverse *Jasminum* species were collected from Flowers Research Institute, Guangxi Academy of Agricultural Sciences and Hengxian Jasmine Flower Research Institute. In order to explore variations within and among species, we collected specimens from seven distinct *Jasminum* species: *Jasminum auriculatum* Vahl, *Jasminum multiflorum* (Burm. f.) Andrews, *Jasminum dichotomum* Vahl, *Jasminum floridum* (Bunge) Banfi, *Jasminum odoratissimum* (L.) Banfi, *Jasminum nudiflorum* Lindl and *Jasminum sambac* (L.) Aiton. Six different accessions of *J. sambac* exhibiting two phenotypes: single-petal and multi-petal, were included in sampling. In total, 12 individual samples were collected for genomic DNA isolation and sequencing in this study. Additional details of sampling refer to Supplementary Table S1. Healthy young leaves were collected, immediately frozen in liquid nitrogen for at least 20 min, and stored at -80 ℃ prior to DNA extraction.

### DNA extraction and sequencing

Total genomic DNAs of 12 individual samples were separately extracted from leaf tissues using a modified cetyltrimethylammonium bromide (CTAB) method [21]. DNA purity and concentration were assessed by a NanoDrop One UV–Vis spectrophotometer (Thermo Fisher Scientific, US). Illumina sequencing of genomic DNAs was performed by Berry Genomics Company (Beijing, China). A paired-end library with a 300–500 bp insert size was constructed using the NEBNext Ultra DNA Library Prep Kit (New England Biolabs, MA, USA) for Illumina, and then subjected to whole-genome resequencing on the Illumina NovaSeq platform (Illumina Inc., CA, USA) in PE 150 nt mode.

### Chloroplast genome assembly and gene annotation

A total of 12 samples of *Jasminum* were used to obtain 18.11–28.93 Gb raw reads with a mean coverage of 36 × to 58 × of whole genomes and 6,960 × to 59,050 × of cp genome base coverage (Table S2). Prior to the de novo assembly of the cp genome, quality control of the raw paired-end reads was performed using Trimmomatic v0.40 [22]. The percentage of bases with a Phred score greater than 30 (Q30) in the overall bases ranged from 90.19% to 92.79%. Then the clean paired-end reads were further used to assemble the cp genomes using NOVOPlasty v4.3.1 software [23] by referencing the published cp genomes of *J. sambac* (GenBank Acc. No. MN158204 and No. MN158205) [24]. To ensure the accuracy, we also employed GetOrganelle v1.7.5 software [25] to thoroughly validate the cp genome assemblies. The software utilizes the seed database to iteratively retrieve target reads, then calls SPAdes for genome assembly. Alignment with the NT database confirms the assembly order of chloroplast contigs, selecting those with a consistent order as the target genome result. Based on the reference genome, the software determines the starting position and direction of chloroplast assembly sequences, as well as the potential partitioning structure of the chloroplast (LSC/IR/SSC), to obtain the final chloroplast genome sequences. The complete cp genomes were then annotated using Geseq [26]. The protein search identity parameter was set at 60 and the rRNA, tRNA, DNA search identity parameter was at 35. We utilized tRNAscan-SE v2.0.7 for tRNA annotation. Based on the preliminary annotation results, the initial redundant genes were removed from the predicted set, and the gene boundaries, as well as exons/introns, were manually corrected to generate a highly accurate gene set. Finally, the circular gene map was visualized using OGDRAW v1.3.1 [27]. The annotated chloroplast genome sequences for the 12 *Jasminum* samples have been submitted to the GenBank database under accession numbers OR730547 to OR730558 (Table S1).

### Codon usage, simple sequence repeats and long repeats analysis

The probability of a specific codon appearing in synonymous codons that encode a specific amino acid can provide insights into the degree of codon usage bias in different species of *Jasminum*. The preference score of codons can be determined through the computation of Relative synonymous codon usage (RSCU). Subsequently, all coding sequences (CDS) were utilized to estimate RSCU using the CUSP program with EMBOSS v6.6.0.0 [28]. An RSCU value above 1.00 indicates an increased frequency of codon usage, while a value below 1.00 suggests a lower frequency of usage than anticipated [29]. Codon Adaptation Index (CAI) was estimated for all coding sequences (CDS) using the CAI program within EMBOSS. The MicroSAtellite (MISA v2.1) [30] identification tool, a perl program, was used to detect simple sequence repeats (SSRs) in the 12 cp genomes. In this study, only perfect repeats were selected for analysis with the following parameters: basic motifs (1–6 bp), a minimum repeat length of 8 bp (for mono-), 10 bp (di-), 12 bp (for tri- and tetra-), 15 bp (for penta-), 18 bp (for hexa-), and a minimum distance of 100 bp between two SSRs. Primer3 (http://www.simgene.com/Primer3, accessed on 10 June 2023) was used to design primers for SSR sequences identified by MISA (https://github.com/declare-lab/MISA, accessed on 14 June 2023). The REPuter V1.0 program [31] was used to identify and map the locations and sizes of forward, reverse, palindrome, and complementary sequences, employing the following parameters: a minimum of 30 bp, a hamming distance of 3, and a maximum of 5,000 computed repeats.

### Comparative chloroplast genome analysis

To determine the sequence divergence among the *Jasminum* cp genomes, the online genome comparison tool mVISTA (https://genome.lbl.gov/vista/index.shtml, accessed on 26 June 2023) was employed, with the *J. sambac* (HTML-8) annotation serving as the reference. The default parameters were configured to align the cp genome in Shufe-LAGAN mode, and the sequence conservation profile was visualized using an mVISTA plot. Furthermore, a comparative analysis of the boundaries of the SSC and IR regions across 11 *Jasminum* species was performed using the IRscope software [32]. DnaSP v5.10 [33] was applied to determine the level of nucleotide diversity (*Pi*) among 12 samples, with the *J. sambac* (HTML-8) cp genome as the standard. When calculating the *Pi* value, both the step size and the sliding window size were set to 650 bp and the same methods were computed to the intraspecific *Pi* values of *J. sambac*. Lastly, the Ka/Ks (non-synonymous/synonymous substitution ratio) values for each protein-coding gene were estimated using the perl script ParaAT v2.0 [34], in combination with muscle v3.8.31 [35] and KaKs_Calculator2.0 [36].

### SNP calling, PCA, and phylogenetic tree construction

The chloroplast genome sequences from the 12 individuals representing 7 species were comparatively analyzed using BWA v0.7.12 [37], employing the -M parameter, with the *J. sambac* cp genome (GenBank Acc. No. MN158205) serving as the reference genome for alignment. The Genome Analysis Toolkit (gatk v4.2.2.0) [38] was used to mark single-sample duplicates. SNP identification was performed by Bcftools mpileup [39]. Subsequently, the vcftools software was employed to retain and filter high-quality SNPs with the following parameters [40]: a maximum missing rate of 0.6, minor allele count (mac) at 3 and minQ at 30. After filtering, 1,179 out of 6,199 possible sites were retained. The EIGENSOFT v7.2.1 package (https://github.com/gurinovich/PopCluster, accessed on 23 June 2023) was used to perform PCA, and EIGENSTRAT [41] was performed on linkage disequilibrium (LD)-pruned pseudomolecule SNPs. The p-distance matrix was calculated using VCF2Dis (v1.47) (https://github.com/BGI-shenzhen/VCF2Dis, accessed on 26 June 2023) with the filtered SNP set obtained. Finally, a neighbor-joining tree was constructed using the UPGMA method. The resulting tree was visualized by iTOL (v6.8.1) [42].

### Phylogenetic analysis based on orthologues

A search was conducted in the NCBI database using the keywords "Oleaceae chloroplast, complete genome" to determine the available cp genomes in Oleaceae and their phylogenetic placement (accessed on 20 May 2023). We downloaded 344 published cp genomes from 25 genus (*Abeliophyllum*, *Chengiodendron*, *Chionanthus*, *Chrysojasminum*, *Comoranthus*, *Fontanesia*, *Forestiera*, *Forsythia*, *Fraxinus*, *Haenianthus*, *Hesperelaea*, *Jasminum*, *Ligustrum*, *Myxopyrum*, *Nestegis*, *Noronhia*, *Notelaea*, *Nyctanthes*, *Olea*, *Osmanthus*, *Phillyrea*, *Picconia*, *Priogymnanthus*, *Schrebera*, and *Syringa*) from NCBI. Incomplete cp genomes or duplicated cp genomes from the same species were then manually removed. Only the most recently published cp genomes of the same species were retained. In the end, we utilized a total of 159 chloroplast genomes for the phylogenetic tree construction, of which 12 were assembled by our team. ORTHOMCL v6.11 [35] was applied to identify orthologous gene families in 159 cp genomes and single-copy orthologues were identified with the BLASTP E-value cut-off of less than 1e^−5^. Using 39 cp single-copy protein-coding genes (*atp*A, *atp*E, *atp*H, *atp*I, *ndh*A, *ndh*C, *ndh*E, *ndh*F, *ndh*G, *ndh*I, *ndh*J, *pet*A, *pet*B, *pet*D, *psa*A, *psa*C, *psb*A, *psb*B, *psb*C, *psb*D, *psb*E, *psb*F, *psb*H, *psb*I, *psb*J, *psb*K, *psb*M, *rbc*L, *rpl*20, *rpl*33, *rpl*36, *rpo*A, *rpo*B, *rpo*C1, *rpo*C2, *rps*16, *rps*18, *rps*2, *rps*4), we reconstructed a phylogenetic tree. Multiple sequence alignments of shared gene datasets were generated with MAFFT v7.487 [43] with default parameters and the ML phylogenetic tree of 39 chloroplast genes was subsequently inferred using IQ-TREE 2 v2.1.4-beta [44]. The most suitable substitution model of ML for 159 samples was assessed to be “Q + F + I + I + R3” according to the Bayesian information criterion (BIC) by the “-m MFP” parameter. Branch supports were calculated using 1,000 ultrafast bootstrap replicates and 1,000 replicates of SH-aLRT test, as specified by the “-alrt” parameter [45].

## Results

### Subsection general features of *Jasminum* complete chloroplast genomes

**Fig. 1:**
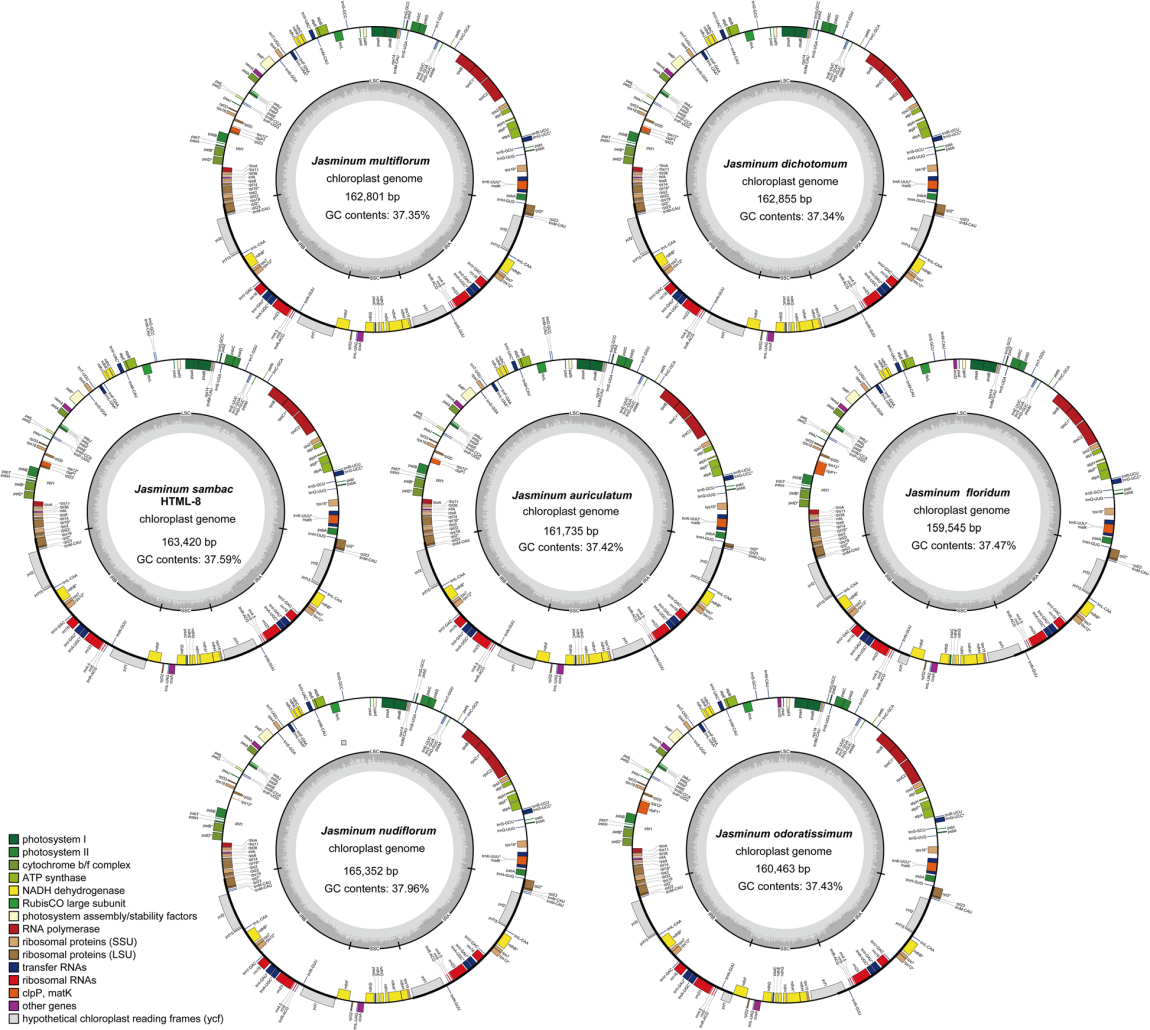
Chloroplast genome maps of seven *Jasminum* species, depicting the GC and AT contents in the inner circle. Functional gene groups are color-coded, with darker gray representing GC content and lighter gray representing AT content

All cp genomes had a circular assembly with a typical quadripartite structure, which was composed of large and small single-copy (LSC and SSC) regions and two inverted repeats (IRs) (Fig. 1, Table 1, and Table S2). The 12 cp genomes ranged from 159,545 to 165,352 bp in length and GC contents varied between 37.34% and 37.96% (Table 1). *J. nudiflorum* had the largest cp genome size, approximately 6 kb longer than that of *J. floridum* cp genome, which was the smallest. The total sizes of *J. sambac* ranged from 163,084 to 163,553 bp for six samples, ranking as the second largest. Despite having the two smallest cp genome sizes, *J. floridum* and *J. odoratissimum* had the longest SSC lengths at 17,703 bp and 17,913 bp, respectively, while *J. sambac* featured the shortest SSC lengths (13,172–13,256 bp). The SSC length of other species fell within the ranges of 13,172 bp to 13,368 bp. In terms of the LSC length, significant variation was observed among the seven species, with *J. dichotomum* having the shortest LSC at 89,611 bp, while *J. nudiflorum* possessed the longest LSC at 92,624 bp.

**Table 1:** Basic chloroplast genome information of 12 samples from *Jasminum*

Taxon	Total Length (bp)	LSC (bp)	SSC (bp)	IR (bp)	Total GC content (%)	Total genes	Portein coding genes	rRNA genes	tRNA gens
*J. auriculatum* EYML-15	161735	91038	13259	57438	37.42	134	87	8	39
*J. multiflorum* MML-9	162801	89713	13368	59720	37.35	134	88	8	38
*J. dichotomum* FBML-17	162855	89611	13354	59890	37.34	134	88	8	38
*J. floridum* TCH-6	159545	91804	17703	50038	37.47	135	88	8	39
*J. odoratissimum* NXML-4	160463	92444	17913	50106	37.43	135	88	8	39
*J. nudiflorum* YCH-12	165352	92624	13256	59472	37.96	134	86	8	40
*J. sambac* XF2H-13	163457	90737	13220	59500	37.58	135	88	8	39
*J. sambac* DSTZML-14	163421	90701	13220	59500	37.59	135	88	8	39
*J. sambac* YNDBML-7	163084	90399	13173	59512	37.56	135	88	8	39
*J. sambac* HTML-8	163420	90700	13220	59500	37.59	135	88	8	39
*J. sambac* CGDBML-11	163475	90801	13172	59502	37.58	135	88	8	39
*J. sambac* JHML-16	163553	90830	13221	59502	37.57	135	88	8	39

**Fig. 2:**
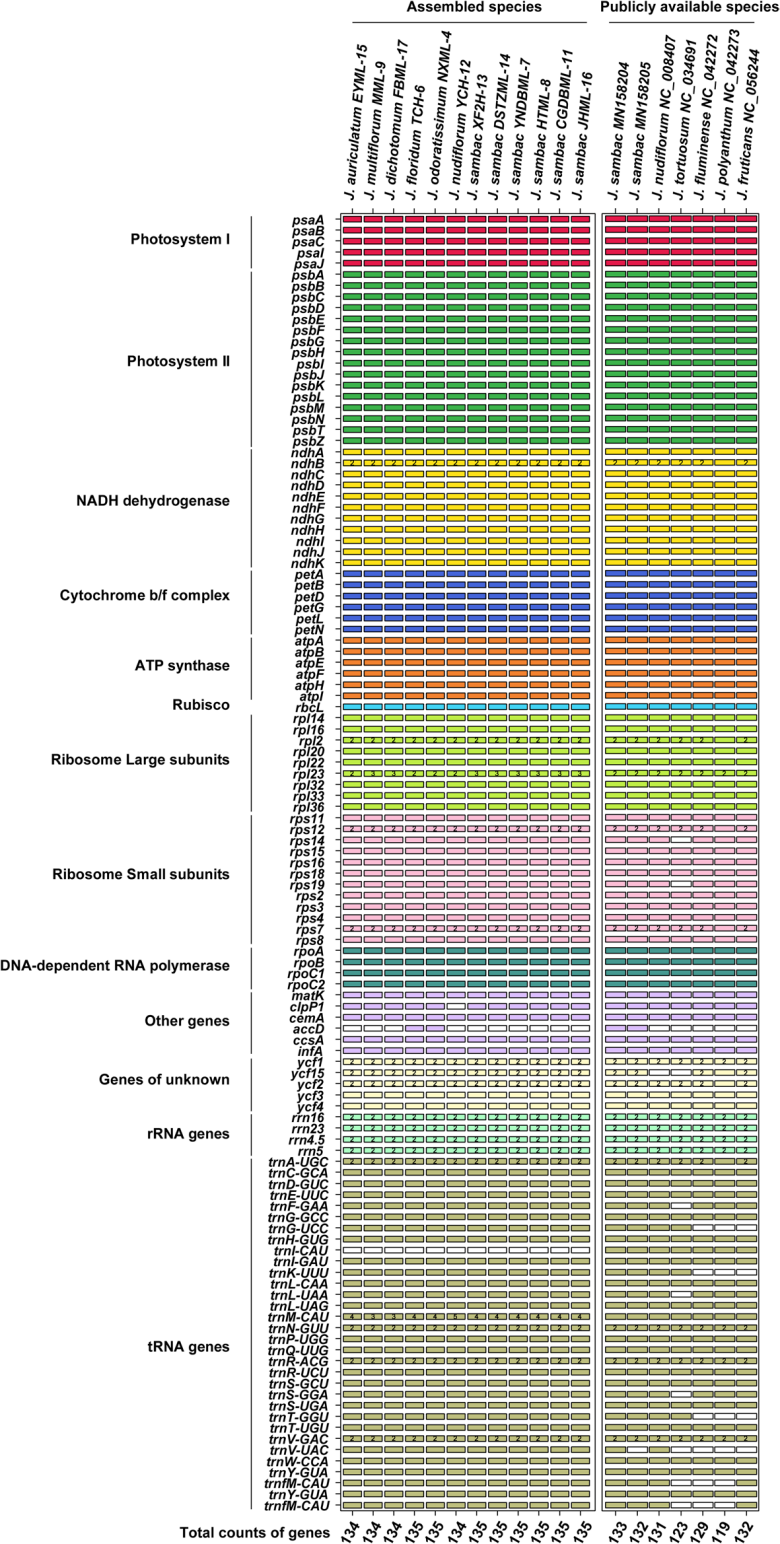
Comparative analysis of gene absence among different *Jasminum* species. Different colors represent the functions of genes, as the white color indicates the absence of genes

In this study, the genomic composition of all twelve *Jasminum* species was similar in cp genomes. The total number of genes varied between 134 and 135 (Fig. 1, Table 1). All samples shared identical sets of eight rRNA genes, and the number of tRNA genes ranged from 38 to 40. The number of protein-coding genes (PCGs) ranged from 86 to 88, specifically with 87 in *J. auriculatum*, 86 in *J. nudiflorum*, and 88 in the remaining species. No differences in the number of genes were observed for Photosystem I & II, NADH dehydrogenase, Cytochrome b/f complex, ATP synthase, Rubisco, DNA-dependent RNA polymerase and rRNA genes (Fig. 2). In the other type of genes, the *clp*P1 gene was only absent in *J. nudiflorum* (YCH-12). *J. floridum* and *J. odoratissimum* had an additional *acc*D gene in our assemblies. In all 12 cp genomes, two copies of 16S-*trn*I-*trn*A-23S-4.5S-5S ribosomal RNA operons were identified in IR regions. These operons were formed by six duplicated genes, comprising four rRNAs (*rrn*4.5, *rrn*5, *rrn*16, and *rrn*23) and two tRNAs (*trn*I*-*GAU and *trn*A*-*UGC). The types of tRNA genes were consistent among the twelve cp genomes, but the variation was observed in the number of tRNA genes (38–40), due to the differing copy numbers of the *trn*M-CAU gene, with three copies in *J. multiflorum* and *J. dichotomum*, five copies in *J. nudiflorum*, and four copies in the remaining nine samples.

Additionally, we obtained seven additional complete *Jasminum* cp genomes from publicly available data in NCBI, and performed a comprehensive comparison of gene types and quantities. When compared to extra downloaded cp genomes, it was intriguing to observe a significant variation in the presence/absence of the *acc*D gene across different *J. sambac* species. In contrast to the other 18 cp genomes, *J. tortuosum* lacked the genes *rps*14, *rps*19, and *ycf*15 (Fig. 2 and Table S3). *J. nudiflorum* (NC_008407) also lacked the gene *ycf*15, while in our study, *J. nudiflorum* (YCH-12) contained *ycf*15 (Fig. 2). In *J. polyanthum*, the *ndh*B, *rps*12, *rps*7, *ycf*2, and *trn*A-UGC genes existed as single-copy genes, whereas in the remaining 18 samples, these genes were duplicated.

### Codon usage analysis

**Fig. 3:**
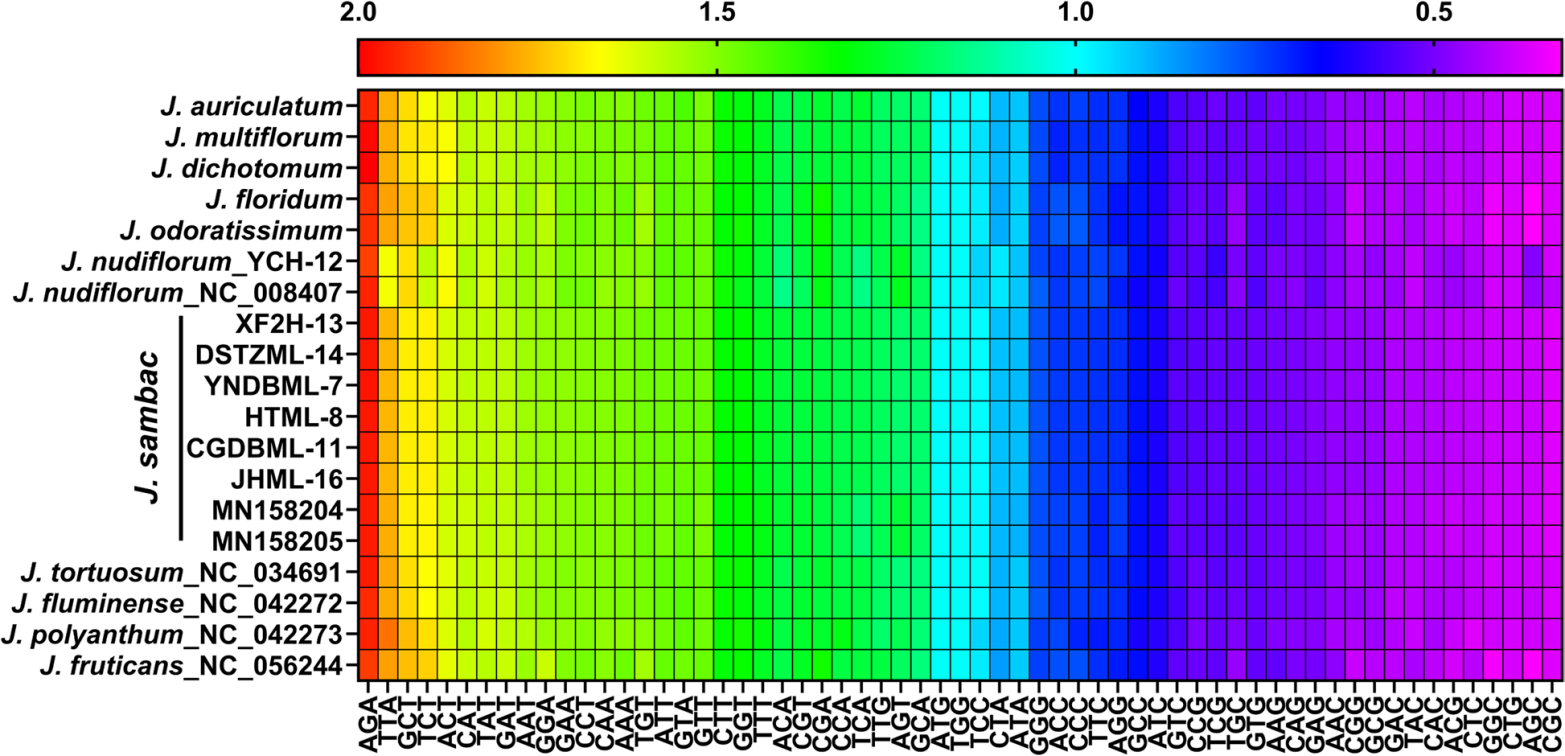
Heatmap illustrating the relative synonymous codon usage (RSCU) values of 19 *Jasminum* species. The color gradient from red to purple represents the range of RSCU values, with red indicating higher values and purple indicating lower values

The relative synonymous codon usage (RSCU) values were computed for *Jasminum* cp genomes based on their protein-coding sequences. Figure 3 shows the codon content of 61 amino acids in all PCGs in the cp genomes of the 11 species (19 cp genomes). In all 19 *Jasminum* cp genomes, *J. polyanthum* has the fewest codons with 24,268, while *J. dichotomum* has the most codons with 27,709 (Table S4). The coding regions of *J. sambac* among eight samples were composed of 27,496 and 27,614 codons. In *J. nudiflorum*, the coding regions were composed of 27,193 and 27,269 codons. The amino acid AGA (Arg) was found to be the most prevalent in *Jasminum* cp genomes, with RSCU values ranging from 1.92 in *J. fruticans* to 2 in *J. dichotomum* (Fig. 3). Conversely, the amino acid CGC (Arg) was the rarest, with RSCU values ranging from 0.36 to 0.41. The usage of the codon TTA (Leu) exhibited variations among different species, with the highest RSCU value of 1.85 in *J. polyanthum* and the lowest value of 1.65 in *J. nudiflorum*. Notably, *J. nudiflorum* displayed significant differences in the usage of codons TCC (Ser), CTA (Leu), and AGC (Ser), with lower RSCU values for TCC (RSCU = 0.94) compared to other species (0.99), while it showed higher RSCU values for CTA (RSCU = 0.96) and AGC (RSCU = 0.47) compared to other species (RSCU = 0.89 and RSCU = 0.37, respectively). In the eight samples of *J. sambac*, the RSCU values of each codon showed minimal variation, with differences not exceeding 0.02. In addition, nearly all A/T-ending codons had RSCU values > 1 in the cp genomes of the 11 species, whereas G/C-ending codons had RSCU values < 1. The potential pattern of polarity or charge for the amino acids corresponding to codons ending in A or T was not detected (see Supplementary Table 8). We calculated the CAI for coding genes but did not identify any significant patterns (see Supplementary Table 9).

### Comparative analysis of repeat elements and SSRs

**Fig. 4:**
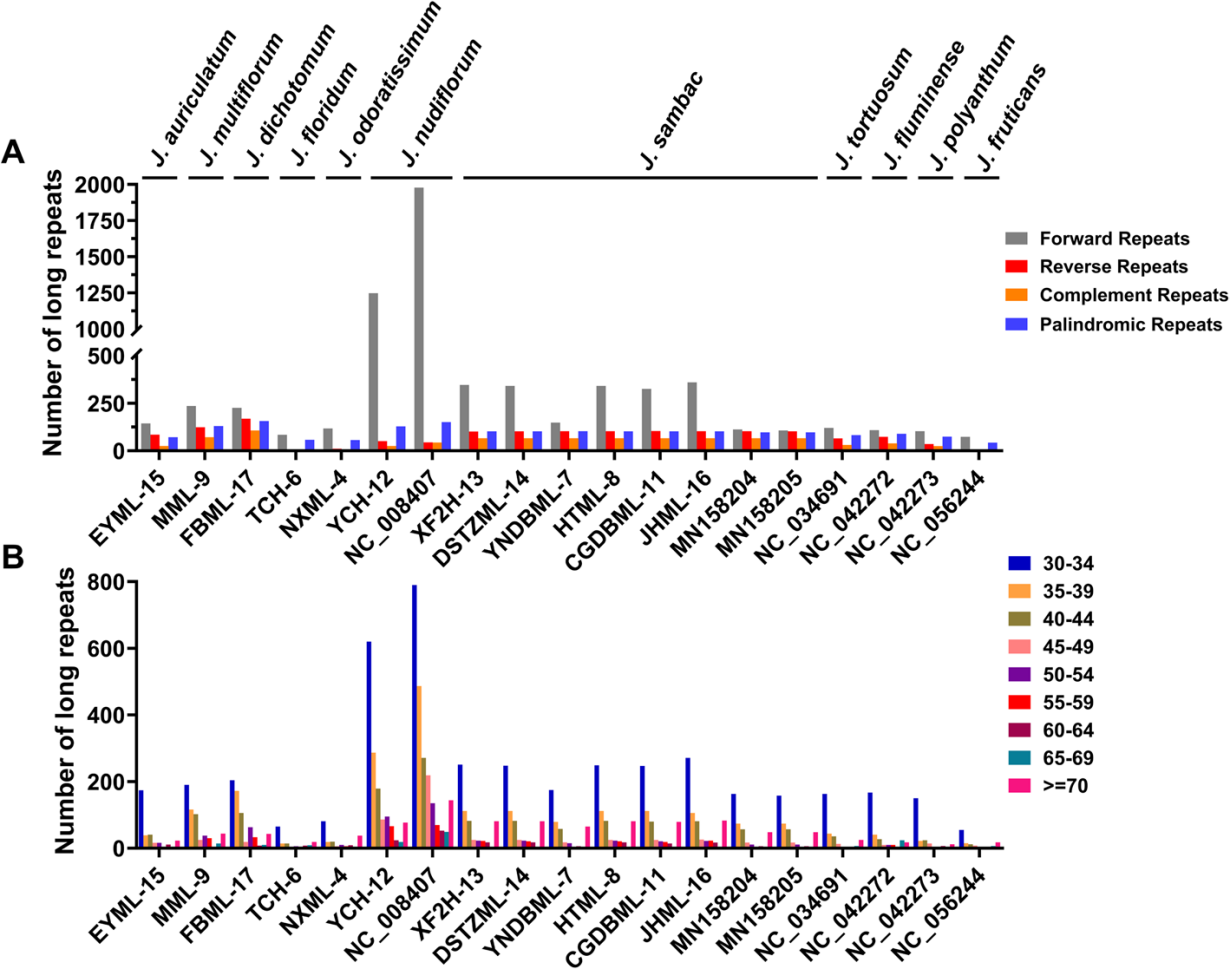
Frequency of various types and lengths of repeated sequences in the chloroplast genomes of 11 species in the *Jasminum* genus. **A** Total count of forward repeats (gray), reverse repeats (red), complement repeats (orange), and palindromic repeats (blue). **B** Total count of repeated sequences categorized by different length intervals. Colors represent distinct length ranges

The distribution of long repeats in *Jasminum* cp genomic sequences was analyzed and summarized (Fig. 4). Four types of repeats were identified: forward repeats (FRs), reverse repeats (RRs), complement repeats (CRs) and palindromic repeats (PRs) (Fig. 4A). The highest number of repeats (2,218) was found in the *J. nudiflorum* cp genome (NC_008407), while the lowest number of repeats (121) was found in the cp genome of *J. fruticans* (NC_056244) (Fig. 4A). A notable discrepancy was observed in the number of FRs among eleven species. The highest number of FRs were found in *J. nudiflorum* (YCH-12) (1,248 FRs) and *J. nudiflorum* (NC_008407) (1,978 FRs), respectively (Fig. 4A). The number of FRs in the other ten species ranged from 73 to 360, significantly lower than that in *J. nudiflorum*. Furthermore, *J. sambac* displayed notable variations in the FRs type among the four repeat categories. Specifically, the *J. sambac* samples MN158204, MN158205, and YNDBML-7 exhibited 112, 107, and 148 FRs, respectively, in their cp genomes, whereas other *J. sambac* cp genomes contained at least 2.2 times more FRs, ranging from 326 to 360. Regarding RRs, the highest abundance of RRs was observed in *J. dichotomum*, totaling 169, followed closely by *J. multiflorum* (124 RRs), and *J. sambac* (101–104 RRs) (Fig. 4B). By contrast, the RRs numbers in *J. floridum* and *J. frutican* were much lower than others, with counts of 3 and 5, respectively (Fig. 4A). In terms of CRs, the number was zero in the *J. floridum* and *J. fruticans* cp genomes, whereas the highest count, 107, was found in *J. dichotomum*. The counts of repeats were classified as nine different groups according to length intervals (Fig. 4B). Within 30–69 bp length range, the maximum number of repeats was observed in the 30–34 bp length group, while the minimum number of repeats was found in 65–69 bp length repeats. *J. nudiflorum* exhibited significantly higher counts of repeats across various length categories compared to other species.

The distribution of six types of SSRs, namely mono-, di-, tri-, tetra-, penta-, and hexa-nucleotide is shown in Figure S1. Most of the SSRs in the 19 cp genomes were found in the LSC region (Fig. S1A). In the SSC region, *J. fruticans*, *J. floridum*, and *J. odoratissimum* had highest numbers of SSRs, with 32, 37, and 38 SSRs, respectively, while the number of SSRs ranging from 14 to 23 in the other species. A significant disparity in SSR distribution among all cp genomes was found in the IR region, with SSR number ranging from 16 to 70. In *J. sambac*, no significant variations were observed in the distribution of SSRs within SSC and IR regions across all eight *J. sambac* samples. The mononucleotide SSR was found to be the most abundant, followed by trinucleotides and hexanucleotide in all 11 species (Fig. S1B). The number of SSRs located in the coding regions was less than half of the entire cp genome (Fig. S1C). Among the SSRs in the coding regions, tri-type SSRs outnumber other length types of SSRs when excluding mononucleotides. Additionally, in contrast to the relatively sparse distribution of hexa-type SSRs in the entire cp genome, their distribution in the coding regions resembled that of other length types of SSRs (Fig. S1B and D). The large number of SSRs detected in this study can serve as potential molecular markers for further research on the *Jasminum* genus plant group (see Table S10, S11, S12, S13, S14, S15, S16, S17, S18, S19, S20, S21).

### *Jasminum* cp genome alignments and IR contraction and expansion

Using the *J. sambac* (HTML-8) cp genome as the reference, the comparative sequence analyses exhibited sequence similarities and gene structure order consistency among the representative cp genomes of eleven different *Jasminum* species (Fig. S2). The findings uncovered a high degree of similarity between the cp genomes of *J. sambac* and ten other species: *J. auriculatum*, *J. multiflorum*, *J. dichotomum*, *J. nudiflorum*, *J. tortuosum*, *J. fluminense*, *J. polyanthum*, *J. floridum*, *J. odoratissimum*, and *J. fruticans*. Among the 11 cp genomes of all species, the variations in the LSC and SSC regions were more pronounced compared to the IR region (Fig. S2). In addition, the coding regions demonstrated minor distinctions in comparison to the non-coding regions, with the 4 rRNA genes being the most conserved regions among all 11 chloroplast genomes. The coding regions that exhibited the most significant differences included *ycf*1, *ycf*2, *psa*I, and *rps*12 (Fig. S2). Sequence alignment analysis across 8 samples of *J*. *sambac* showed that MN158204 and MN158205 exhibited noticeable variation in position from 46 to 66 kb when compared to the reference HTML-8, indicating the absence of *acc*D gene (Fig. 2, Fig. S3, and Table S3).

**Fig. 5:**
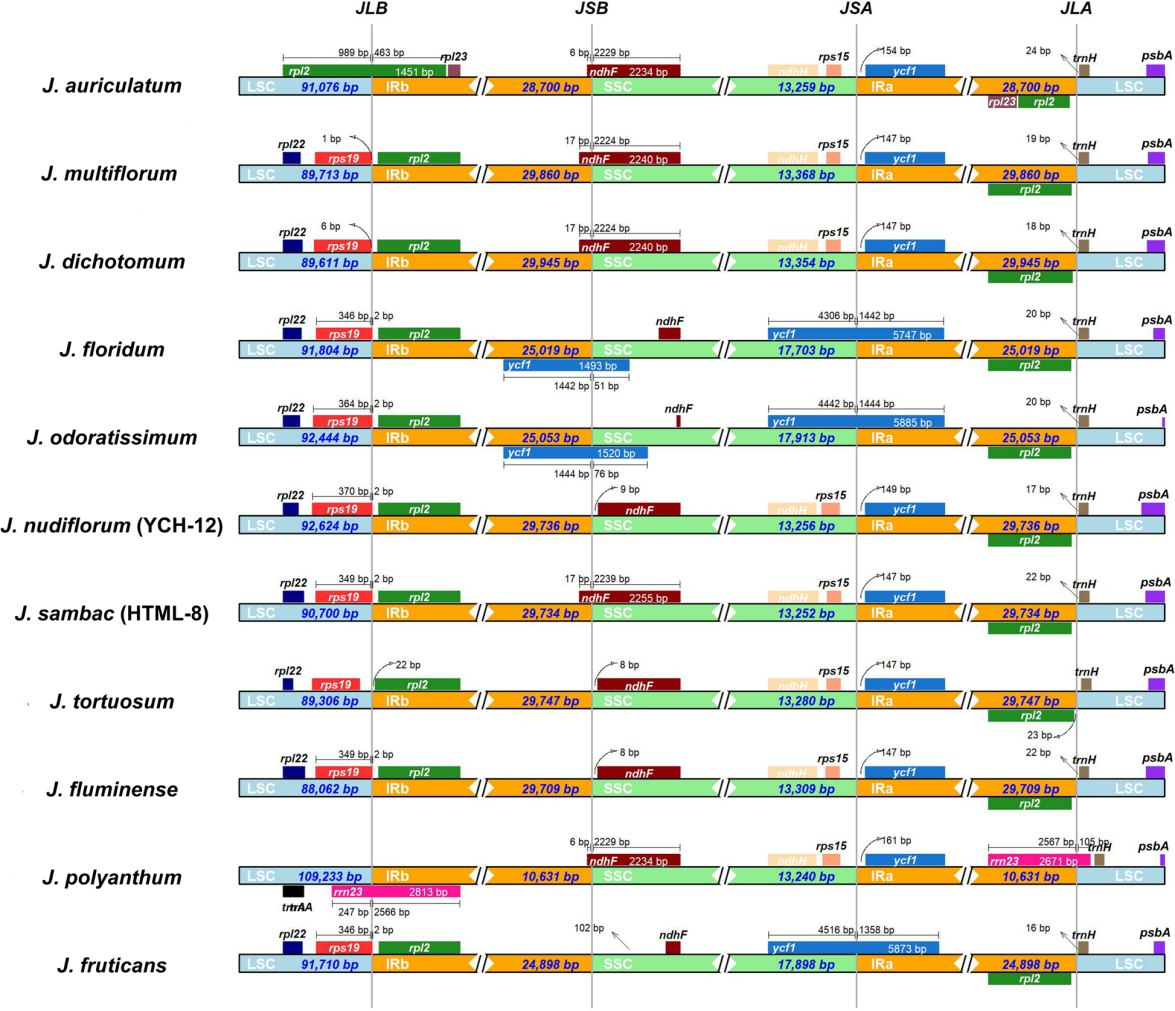
Comparison of the borders of LSC, SSC, and IR regions in 11 chloroplast genomes of the *Jasminum* genus. Genes or gene segments are highlighted in color boxes on both sides of the junctions. The numbers above or below the gene features represent the distance between the gene ends and the junction sites, with arrows indicating the location of the distance

In the chloroplast genomes of 11 different species of *Jasminum*, a general trend of conservation in the borders of IRa/SSC, IRa/LSC, and IRb/LSC. The IRa/SSC junction was commonly located between the *ycf*1 and *rps*15 genes in eight *Jasminum* species (Fig. 5). In contrast, in *J. floridum*, *J. odoratissimum*, and *J. fruticans*, the *ycf*1 gene extended beyond the IRa/SSC border. The IRb/LSC border was typically located between the *rpl*2 and *rps*19 genes in most (9/11) samples. However, in *J. auriculatum* and *J. polyanthum*, the border was crossed by the *rpl*2 gene and *rrn*23 gene, respectively. The IRb/SSC junction in chloroplast genomes showed great variation among *Jasminum* species, which could be divided into three categories. In *J. floridum* and *J. odoratissimum*, IRb/SSC border was located within the *ycf*1 gene. In *J. auriculatum*, *J. multiflorum*, *J. dichotomum*, *J. sambac*, and *J. polyanthum*, the IRb/SSC border was located within the *ndh*F gene. In the remaining four species, *J. nudiflorum*, *J. tortuosum*, and *J. fluminense*, the IRb/SSC border was situated 8–9 bp from the *ndh*F gene, while in *J. fruticans*, the IRb/SSC border was located 102 bp away from the *ndh*F.

### Hotspots of sequence divergence in *Jasminum* cp genomes and selective pressure analysis

**Fig. 6:**
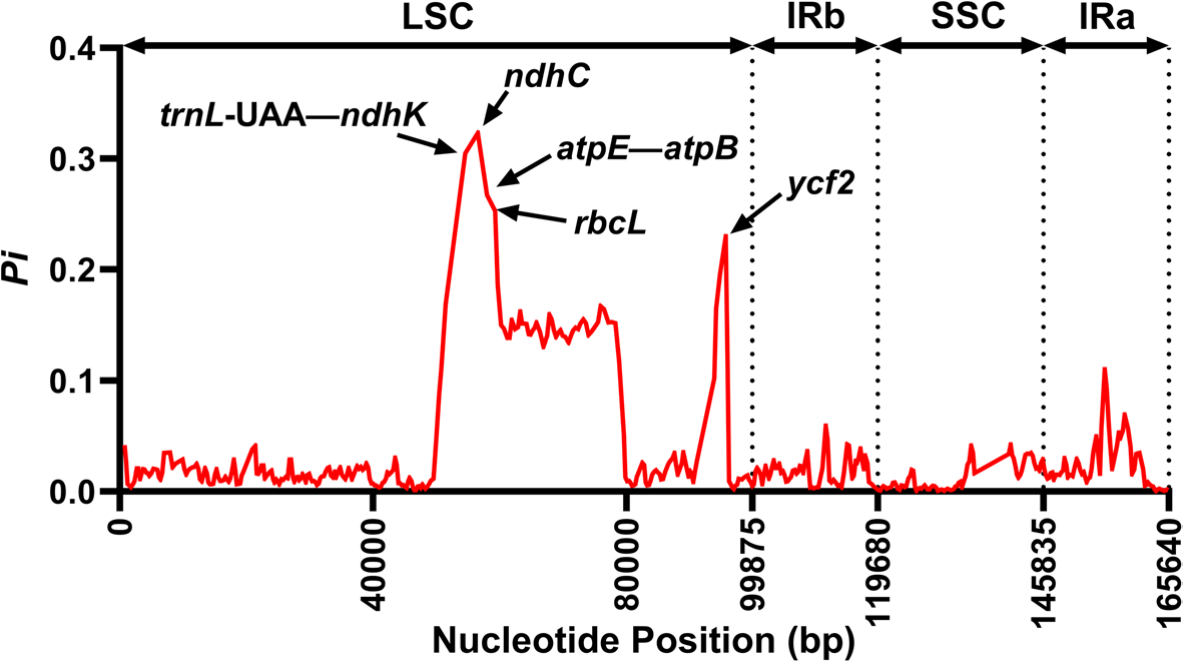
The nucleotide diversity (*Pi*) value in 500 bp sliding-window of the 19 *Jasminum* whole chloroplast genomes. The genes annotated indicate high *Pi* value (over 0.2)

The nucleotide diversity (*Pi*) value was calculated using the DnaSP program to evaluate the mutation hotspots in the 19 cp genomes of eleven species (Fig. 6). The results illustrated that the *Pi* values varied from 0 to 0.32 in the peer window of all 19 *Jasminum* cp genomes (Table S5 and Fig. 6). Five of these loci, *ycf*2 (0.23), *rbc*L (0.25), *atp*E (0.27), *ndh*K (0.30), and *ndh*C (0.32), showed the highest values (*Pi* > 0.2). In the IRa, IRb, and SSC regions, the *pi* values ranged from 0 to 0.1, whereas the LSC region exhibited more extensive and elevated *pi* values, varying from 0 to 0.32.

**Fig. 7:**
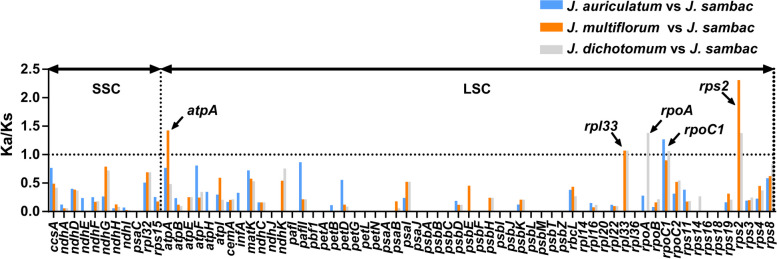
Ka/Ks ratio of 70 protein-coding genes in *J. auriculatum, J. multiflorum* and *J. dichotomum* compared with *J. sambac* chloroplast genomes. Comparisons between different species are represented by different color bars. Blue boxes indicate the Ka/Ks ratio for *J. auriculatum* vs *J. sambac*; orange, *J. multiflorum* vs *J. sambac*; gray, *J. dichotomum* vs *J. sambac*. Annotated genes indicate the corresponding comparative Ka/Ks ratio exceeds one

To investigate the genetic selection differences and explore genome evolution between them, Ka/Ks values were calculated and compared for 70 PCGs in *J. sambac* in comparison to each gene of the three most closely related species (*J. auriculatum*, *J. multiflorum* and *J. dichotomum*). The average Ka/Ks value ratio for the 70 PCGs was slightly higher (mean Ka/Ks = 0.23 ± 0.14711) in the comparison of cp genomes between *J. multiflorum* and *J. sambac*, followed by the group of *J. dichotomum* vs. *J. sambac* (mean Ka/Ks = 0.22 ± 0.10661) and the group of *J. auriculatum* and *J. sambac* (mean Ka/Ks = 0.19 ± 0.06960) (Table S7). Among the 70 PCGs, the Ka/Ks values for five genes, *rps*2, *atp*A, *rpo*A, *rpo*C1, and *rpl*33, were found to be highest (Fig. 7). The *rpo*C1 gene exhibited relatively high Ka/Ks values in all three comparisons, with values of 1.27 (*J. auriculatum* vs *J. sambac*), 0.90 (*J. multiflorum* vs *J. sambac*), and 1.06 (*J. dichotomum* vs *J. sambac*). In the comparison of *J. multiflorum* vs *J. sambac*, the *rps*2 gene displayed the highest Ka/Ks value of 2.31, followed by the *atp*A gene (1.42), and the *rpl*33 gene (1.07). In *J. dichotomum* vs *J. sambac*, *rpo*A gene presented the highest Ka/Ks value of 1.38, followed by *rps*2 (1.37), *rpl*33 (1.07) and *rpo*C1 (1.06). The *rpo*C1 gene was the only one with a Ka/Ks value greater than 1 (1.27) in the group of *J. auriculatum* and *J. sambac*.

### SNP identification and structure analysis among *Jasminum* cp genomes

**Fig. 8:**
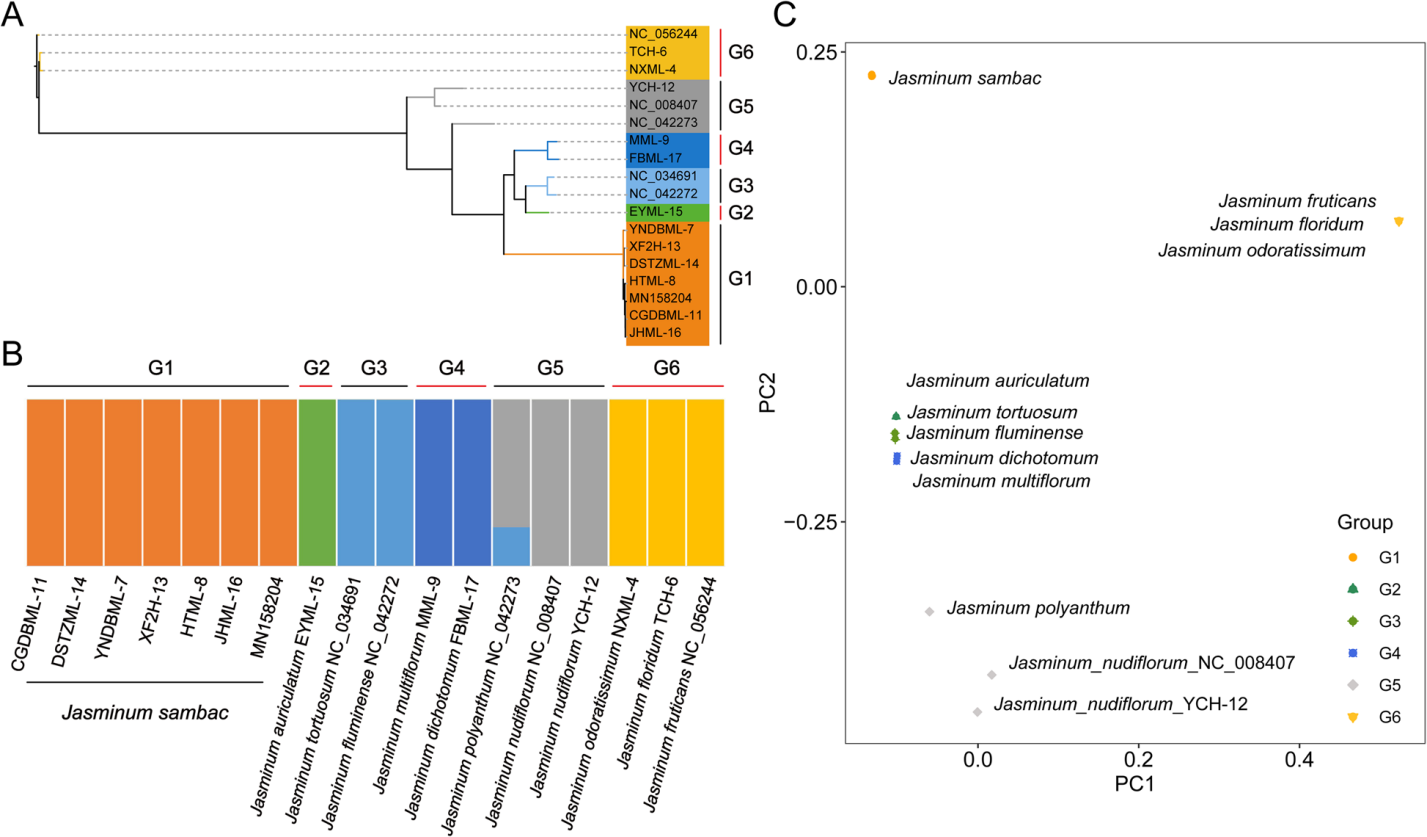
Analysis of chloroplast genomes SNPs in the *Jasminum* genus. **A** Phylogenetic analysis results based on 18 samples of the *Jasminum* genus, categorized into six groups labeled as G1 to G6. **B** Structure analysis results based on 18 *Jasminum* samples, showing six groups that correspond with the phylogenetic tree. Different colors indicate distinct gene flow signals corresponding to the groups. **C** Principal component analysis (PCA) results based on 18 *Jasminum* chloroplast genomes, also dividing the samples into six groups labeled as G1 to G6, which correspond with the phylogenetic tree and Structure analysis

The cp genomes of 19 samples representing 11 species of *Jasminum* were analyzed using SNP identification. The reference genome used here was the cp genome of *J. sambac* (GenBank Acc. No. MN158205.1) downloaded from NCBI. The high-quality SNP data obtained from this analysis was used to construct the phylogenetic tree of all the 18 samples, which revealed the presence of six distinct clusters, labeled as G1 to G6 (Fig. 8A). Within these clusters, the seven *J. sambac* samples formed a single group, G1 (Fig. 8). Another branch consisted of five species that could be divided into G2 (*J. auriculatum*), G3 (*J. tortuosum* and *J. fluminense*), and G4 (*J. multiflorum* and *J. dichotomum*). The G5 group comprised two samples from *J. nudiflorum* (YCH-12 and NC_008407) along with *J. polyanthum*. The remaining three species, *J. odoratissimum*, *J. floridum*, and *J. fruticans*, constituted the outgroup G6. The results of the structure analysis (K = 6) were consistent with the phylogenetic tree (Fig. 8B). The structure analysis revealed a pattern of relatively independent gene flow among species, corresponding to the G1 to G6 clusters observed in the phylogenetic tree. Additionally, the relationships among the 18 samples were analyzed using the principal component analysis (PCA) (Fig. 8C). The result further confirmed that these 18 samples were grouped into 6 clusters, aligning with the patterns observed in both the phylogenetic tree and structure analysis.

A total of 1,179 high quality variations were identified in the 18 *Jasminum* cp genomes when compare to the *J. sambac* reference cp genome, comprising 1,125 (95.24%) SNPs and 54 (4.76%) InDels (Fig. S4A). In total, 33.16% (391) of the variations were present in intergenic space regions, while 6.36% (75) of the variations were located in exons and 60.48% (713) in the introns (Fig. S4B). The distribution of InDels with different lengths were shown in Figure S4C. The largest InDel mutation, spanning 139 bp (Fig. S5), was observed in the spacer region between *trn*N (GUU) and *trn*R (ACG) in three species *J. odoratissimum*, *J. floridum*, and *J. fruticans*. The original sequence underwent a deletion mutation resulting in the replacement of "T" in these species. These three species form the G6 group in the results of the phylogenetic tree, structure analysis, and principal component analysis (PCA) (Fig. 8). The second longest InDel mutation, spanning 75 bp (Fig. S5), was observed between the *trn*V-GAC and *trn*I-GAU genes in seven species (*J. dichotomum*, *J. fluminense*, *J. fruticans*, *J. tortuosum*, *J. odoratissimum*, *J. floridum*, and *J. nudiflorum*). In the phylogenetic tree, structure analysis, and PCA results, *J. fluminense* and *J. tortuosum* formed the G3 group, while *J. odoratissimum*, *J. floridum*, and *J. fruticans* formed the G6 group (Fig. 8). The third longest InDel, spanning 51 bp (Fig. S5), was observed between the *ccs*A and *ndh*D genes in the species *J. fluminense, J. fruticans*, *J. odoratissimum*, and *J. floridum*.

### Phylogenetic relationships of Oleaceae based on complete chloroplast genomes

**Fig. 9:**
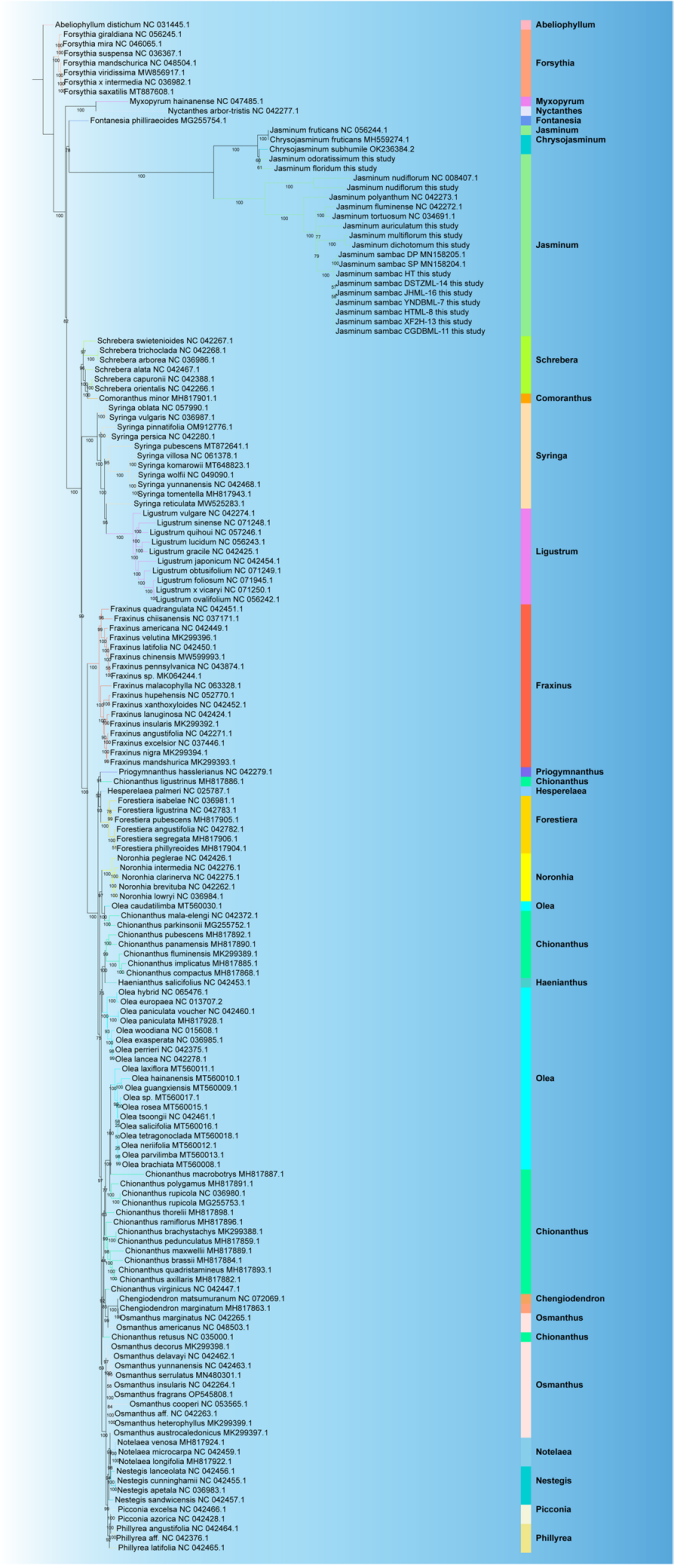
Maximum-likelihood (ML) phylogenomic tree constructed with 159 chloroplast genomes from selected chloroplast of 25 genus (*Abeliophyllum, Chengiodendron, Chionanthus, Chrysojasminum, Comoranthus, Fontanesia, Forestiera, Forsythia, Fraxinus, Haenianthus, Hesperelaea, Jasminum, Ligustrum, Myxopyrum, Nestegis, Noronhia, Notelaea, Nyctanthes, Olea, Osmanthus, Phillyrea, Picconia, Priogymnanthus, Schrebera,* and *Syringa*). Bootstrap (BS) values (1,000 replicates) are indicated at nodes. Complete chloroplast genome sequences were retrieved and downloaded from GenBank and GenBank accession numbers were listed next to their corresponding species. Scale bar represents substitutions per site

To determine the phylogenetic relationships within the *Jasminum* genus, we compared complete cp genomes from 159 samples and constructed a maximum-likelihood (ML) phylogenetic tree for Oleaceae using IQ-TREE, employing 39 shared single-copy genes (Fig. 9). All 19 *Jasminum* cp genomes were included, consisting of 12 cp genomes assembled in this study, along with 7 obtained from NCBI. *Abeliophyllum* was used as an outgroup and rooted the tree. The first divergent branch consisted of the *Forsythia* genus, located as the basal lineage, where all seven different species of this genus clustered together. The next branch to diverge included the *Myxopyrum* and *Nyctanthes* genera, which formed a sister relationship and clustered together. As indicated in abovementioned SNP analysis (Fig. 8), individuals from *J. sambac* formed a distinct clade, whereas the other species within the *Jasminum* genus mainly clustered into five separate clades. It is noteworthy that the *Chrysojasminum* genus has merged with the G6 group (*J. odoratissimum*, *J. floridum*, and *J. fruticans*) into a monophyletic clade within the *Jasminum*. The *Fontanesia* genus, represented by *Fontanesia philliraeoides*, formed a single branch as the closest relatives and outgroup to *Jasminum* and *Chrysojasminum*. Eleven other representative genera, including *Schrebera*, *Syringa*, *Ligustrum*, *Fraxinus*, *Forestiera*, *Noronhia*, *Chengiodendron*, *Notelaea*, *Nestegis*, *Picconia*, and *Phillyrea*, each containing 2–17 distinct species, exhibited clear and independent clustering within their respective genera. By contrast, the relationships within the *Chionanthus*, *Osmanthus*, and *Olea* genera exhibited a higher level of complexity. Notably, *Chionanthus retusus* was found to cluster within the *Osmanthus* genus, while *Osmanthus caudatilimba* clustered with *Chionanthus*. Numerous species of the *Olea* genus have independently formed distinct branches. In terms of the 19 *Jasminum* samples, this result aligned with the phylogenetic trees constructed with SNPs (Fig. 8A) and 39 shared single-copy genes in these cp genomes (Fig. 9).

## Discussion

### Genome features and codon usage

The chloroplast genome size of angiosperms varies from 120 to 180 kb, with an IR region of 20 to 30 kb in length [46]. The complete chloroplast genome consists of a single circular molecule with four distinct regions, separated by the LSC and SSC regions, and two IR regions [47]. In this study, the chloroplast genome sizes of 12 *Jasminum* samples from the Oleaceae family ranged from 159 to 165 kb, while an IR region length of 25 to 29 kb (Fig. 1 and Table 1), which is consistent with the typical chloroplast genome size in angiosperms. The differences in genome size among the 12 *Jasminum* samples were approximately 7 kb to 9 kb in magnitude (Table 1), with the IR region showing the greatest difference (9.6 kb), followed by the SSC region (4.7 kb) and the LSC region (1.4 kb). This indicates that the variation in genome size among different *Jasminum* species is primarily due to differences in the IR region. Previous studies have shown that the presence of an IR enhances the stability and conservation of the chloroplast genome [48]. No reports have been documented concerning excessively prolonged, abbreviated, or absent IR regions in Oleaceae and *Jasminum* cp genomes, which have been observed in *Cryptomeria japonica*, *Erodium texanum*, *Geranium palmatum*, *Monsonia speciosa* and *Pelargonium* × *hortorum* [15, 49–51]. Although no disparities were observed in the presence or absence of genes in the IR region of the chloroplast genomes of the 11 *Jasminum* species, we identified a distinct pattern in the *rpl*23 and *trn*M-GUU genes within the IR region. Notably, these genes not only exist in two copies but also exhibit variations in copy numbers within the LSC region (Fig. 2 and Table S3). In the comparison of chloroplast genome structure and sequences, it was interesting to observe that HTML-8, MN158204, and MN158205 of *J. sambac* exhibited distinct sequences from position 46 to 66 kb, with the absence of the *acc*D gene (Fig. S3). The loss of the *acc*D gene has been reported to be associated with cp genome rearrangements and the acceleration of gene relocation [48, 52–55], potentially causing divergence in different *Jasminum* species. The *acc*D gene encodes the beta subunit of acetyl-CoA carboxylase in the chloroplast. Acetyl-CoA carboxylase is an important enzyme in the chloroplast involved in the carboxylation reaction in the fatty acid synthesis pathway. It is noteworthy that the absence of the *acc*D gene is linked with rearrangement hotspots where it has been lost. The absence of the accD gene could potentially accelerate gene relocations through unknown mechanisms or induce sequential changes through various gene movements [54]. Another discovery is that in *Trifolium*, the *acc*D gene has been transferred to the nuclear genome [56]. The function of *acc*D has been replaced by nuclear copies of an *acc*D-like gene in *Pedicularis* spp [49].

Factors affecting codon usage vary among different plant species. Genome nucleotide mutation bias is considered a primary cause of codon bias in seed plants [57]. In this study, A/T-ending codons generally had RSCU values greater than 1, while G/C-ending codons had RSCU values less than 1 (Fig. 3), indicating a bias towards A/T-rich codons in the *Jasminum* chloroplast genomes. The RSCU values of codons in *J. sambac* showed minimal variation among the eight samples. Previous studies found that in *J. sambac*, 96.7% (29/30) of the preferred synonymous codons end with A/U, while 90.6% (29/32) of the nonpreferred synonymous codons end wsith G/C [15]. The predominant usage and frequency of A or T-ending codons, along with the lower preference for codons ending in G or C, are major factors that influence the codon usage bias of *Jasminum* chloroplast genes. The genomes of plant chloroplasts usually exhibit an AT bias, as seen in *Camellia* [58], *H. davidii* [59], *Gynostemma* [60], *Asteraceae* [61], and *M. chinensis* [62]. This tendency may be associated with enhancing gene expression. The chloroplast genome may have undergone selection pressure during evolution, leading to adaptations for specific replication and transcription mechanisms that favor the utilization of AT bases. However, we did not detect any discernible pattern of polarity or charge for the amino acids corresponding to codons ending in A or T (see Supplementary Table S8).

These observations indicate that there are preferences and variations in codon usage among different *Jasminum* species, potentially influenced by factors such as evolutionary history, selection pressure, and genomic composition. Further studies are needed to investigate the functional implications of these codon usage patterns in *Jasminum* species.

### Repeat and SSR characteristics

Repeat regions play a crucial role in genome recombination and rearrangement, with the copy numbers varying among different species and even within the same species [63, 64]. Long and complex repeat sequences may have significant implications in genome rearrangement or recombination [65, 66], and recombination between repeat sequences can induce genome rearrangement [67]. In *J. sambac*, we observed that all the six complete chloroplast genomes of *J. sambac* had the same number of CRs, but the other three types of repeats (FRs, PRs, RRs) differed. Particularly, *J. sambac* YNDBML-7 had only 148 forward repeats, whereas the other five cp genomes had 341–360 FRs (Fig. 4). The number of FRs in YNDBML-7 is consistent with previous studies (107 and 112) [68]. In *J. nudiflorum*, significant differences in repeat sequence numbers were found compared to other species, with 1,248 FRs, approximately 4–6 times more than the other species in this study (Fig. 4A). Repetitive sequences may lead to genome rearrangements, thereby promoting genetic differentiation of the genome. There is no consistent pattern regarding the prevalence of specific types of repetitive sequences across various species. For instance, significant variations in the number of FRs were also observed among different *Commiphora* species [69]. By contrast in some other species such as *Euphoria* and *Teucrium*, no notable differences were observed in FRs; instead, TRs were more abundant [70, 71]. Regarding RRs and CRs, they were relatively scarce in *J. nudiflorum*, about half of the other species (except *J. fruticans* and *J. floridum*). *J. fruticans* and *J. floridum* had the lowest number and types of repeat sequences and no complement repeats. The relatively low abundance of RRs and CRs in *J. nudiflorum*, *J. floridum*, and *J. fruticans* may be related to the genome structure and evolutionary history. *J. floridum*, and *J. fruticans* were the first species to diverge from Fontanesia, forming a dinstinct group within *Jasminum* (Fig. 9)*,* which can explain their relatively limited number and variety of repeats. *J. nudiflorum* nested within the subsequent group that diverge from the first one (Fig. 9). Then, the other Jasmine species in the next distinct cluster may have undergone evolutionary processes involving genome reshaping, leading to an expansion in RRs and CRs due to the influence of evolutionary pressures. The presence of repetitive sequences may increase the size and complexity of the *Jasminum* chloroplast genomes. Plant species may have undergone evolutionary processes to expand/reduce repetitive sequences to optimize genome structure and function. The reduction of repetitive sequences may contribute to the genome stability.

We conducted a thorough comparison of the distribution of SSRs in the 19 cp genomes of *Jasminum*, including their presence in various regions: LSC/SSC, IR, coding regions, as well as the entire genome. The total number of SSRs showed no significant difference (Fig. S1A), consistent with the findings in Oleaceae [15]. In the coding region, mono-type SSRs stood out as the most abundant, with tri- consistently ranking as the second most prevalent in each species (Fig. S1D). Trinucleotide repeats have been observed as the most abundant in various species, including citrus (*Citrus reticulata Blanco*) [72], *Psammosilene tunicoides* [73], *Codonopsis pilosula* [74] and mango (*Mangifera indica L*) [75]. The advantage of trinucleotide and hexanucleotide repeats over other types of repeats is attributed to negative selection against frameshift mutations. Tri- and hexa-nucleotides incorporate multiple codons, and their mutations may avoid disrupting the reading frame, thereby contributing to the preservation of genetic function [76]. The utilization of SSRs in the construction of genetic linkage maps, identification of varieties, and development of molecular markers has been well established [77, 78]. The detailed information about the identified SSRs in this study can facilitate future research on selected target regions, allowing for more in-depth population studies among the eleven species within the *Jasminum* genus. Also, the abundant repeat sequences in the chloroplast genomes of *Jasminum* species may hold significant implications for genome stability and evolution.

### IR contraction, expansion, and high-divergent regions

The evolution of cp genomes often involves recurring events such as gene loss [79], sequence inversion [64], and contraction and expansion at the borders of the LSC, SSC, and IR regions [80, 81]. In many other species, the *ycf*1 gene has been recognized as a pseudogene located within the boundary regions between IRb and SSC, and partial gene duplication has led to a loss of protein-coding ability in *ycf*1 [80, 82]. However, in this study, *ycf*1 did not extend across the boundary region between IR and SSC in the majority of *Jasminum* species, except for *J. floridum, J. odoratissimum,* and *J. fruticans*. Both the IRb/SSC and IRa/SSC boundaries of *J. floridum* and *J. odoratissimum* were situated within the *ycf*1 gene. The IRa/SSC boundary of *J. fruticans* was located within *ycf*1. In the case of the remaining species, no *ycf*1 gene overlap was observed at the boundary between IR and SSC (Fig. 5). Additionally, we observed that the LSC/IRb boundaries in most (9/11) *Jasminum* species were located between the *rps*19 and *rpl*2 genes, with varying distances from the LSC/IRb boundary, ranging from 2 to 22 bp in IRb and 1 to 349 bp in LSC. In other two species, the LSC/IRb boundary was located within the *rpl*2 gene in *J. auriculatum* and the *rrn*23 gene in *J. polyanthum*. The IRb/SSC border is located within or near the *ndh*F gene in 8 out of 11 *Jasminum* species. These findings indicate that variations in the expansion and contraction of the IR region are common in *Jasminum* cp genomes, providing insights into the evolutionary relationships and genomic structures within this genus.

Although cp genomes are considered conservative among angiosperm species, high-divergent regions can be observed even among closely related species [83, 84]. Here, we identified five genes (*ycf*2, *rbc*L, *atp*E, *ndh*K, and *ndh*C) as high-divergent regions within the LSC region (*Pi* > 0.2) (Fig. 6). This aligns with previous findings indicating that the IR regions in the cp genome remain conserved and stable due to the copy-dependent repair mechanism, resulting in less variation compared to the LSC and SSC regions [48, 85]. The genes *ndh*K and *ndh*C are often identified as hotspots of variation in the chloroplast genome. These genes have been located in diversity hotspots in plants such as white oak [86], Leguminosae [87], and Ranunculaceae [88]. These sites hold significance as potential molecular markers for uncovering close relationships. The phylogenetic tree revealed that the *J. sambac* clade exhibited a sisterly relationship with the *J. auriculatum*, *J. multiflorum*, and *J. dichotomum* clades (Fig. 9). The Ka/Ks values for certain genes in the cp genomes of these four species indicated that positive selection acted on genes such as *rps*2, *atp*A, *rpo*A, *rpo*C1, and *rpl*33 (Fig. 7). These genes may play crucial roles in biological processes such as photosynthesis and energy metabolism in the chloroplast genome [89].

### Evolutionary history of Oleaceae

The comprehensive analysis of the chloroplast genome confers a distinct advantage in elucidating the phylogenetic relationships within extensive and intricate plant lineages [90]. In this study, a phylogenetic tree was constructed based on the shared 39 PCGs found in the chloroplast genomes of 159 samples (representing 149 species) across 25 genera within the *Oleaceae* family (Fig. 9). Most species from the same genus clustered together with robust support in this phylogenetic tree, with two closely related species *A. distichum* and *F. giraldiana* at the base of the evolutionary tree, representing their primitive position within the Oleaceae family. This observation was corroborated by the phylogenetic analysis involving *rps*16 and *trn*L-F sequences (Wallander and Albert, 2000), as well as by the analysis with *ndh*F and *rbc*L, both of which indicated that *Abeliophyllum* and *Forsythia* constituted basal lineages [54].

Our study strongly suggests the monophyletic nature of chloroplast genomes within the genus *Jasminum*. This finding is in line with other studies indicating that *Jasminum* forms a monophyletic group and its divergence commenced in the Late Cretaceous (78.3 MYA), with certain species of the genus diverging in the Middle Eocene (42.1 MYA) [54]. The proposed existence of a "ghost lineage" sister to Jasmineae, which is likely the maternal parent of the tribe Oleeae, suggests that the ancestral lineage of Jasmineae may not be the direct ancestor [17]. The clustering of *Jasminum* species in our results supports the previous classification of *Jasminum* into five sections: *Alternifolia*, *Unifoliolata*, *Jasminum*, *Primulina*, and *Trifoliolata* [17], strongly questioning the existing morphological classification [18]. This tree incorporated additional species of *Jasminum*, unveiling that *J. auriculatum*, *J. multiflorum*, and *J. dichotomum* were the species most closely related to *J. sambac*. This is inconsistent with the previous tree of *Jasminum* constructed based on representative cpDNA genes (*trn*L-*trn*F spacer, *mat*K, and *psb*A-*trn*H), showing that *J. nudiflorum* stood as the nearest species to *J. sambac* [68]. According to the evolutionary tree, as well as the results from PCA and structure analysis of SNPs from 18 samples, we found that *J. fruticans* formed a distinct G6 group with *J. floridum* and *J. odoratissimum* (Fig. 8). Its positioning within the phylogenetic framework of 159 species in the Oleaceae family showed minor discrepancies (Fig. 9). In this extensive lineage, *J. fruticans* initially clustered with *Chrysojasminum subhumile* before associating with *J. floridum* and *J. odoratissimum*. Overall, the currently available data together with the complete cp gene set in our study depict the most comprehensive phylogenetic tree for *Jasminum* to date.

In conclusion, our study provides new insights into the phylogenetic relationships within the Oleaceae family, with specific emphasis on the genus *Jasminum*. The utilization of complete chloroplast genomes and the inclusion of a broader range of species have improved the resolution and accuracy of the phylogenetic analysis. Further studies are needed to explore the evolutionary trajectory and diversification of these plant lineages.

## Conclusions

In this study, we analyzed 19 chloroplast genomes of 11 species belonging to *Jasminum* within the Oleaceae family, consisting of 12 newly assembled cp genomes and 7 obtained from NCBI. The structural and general features of the cp genomes, together with the comparative analysis among different cp genomes, offered new insight into the relationships and evolution within the *Jasminum* genus and among the Oleaceae species. The phylogenetic tree strongly suggested the monophyletic nature of cp genomes within the genus *Jasminum*. Overall, multi-species cp genome analysis of *Jasminum* provides comprehensive perspectives on its genetic diversity and evolutionary history.

### Supplementary Information

**Supplementary Material 1.**

**Supplementary Material 2.**

## Data Availability

The annotated chloroplast genome sequence data that support the findings of this study are openly available in GenBank of NCBI (Accession No. from OR730547 to OR730558). All relevant data can be found in the manuscript and its supplementary materials.

## Abbreviations

*J. sambac*: *Jasminum sambac*
*J. auriculatum*: *Jasminum auriculatum*
*J. nudiflorum*: *Jasminum nudiflorum*
*J. floridum*: *Jasminum floridum*
*J. multiflorum*: *Jasminum multiflorum*
*J. dichotomum*: *Jasminum dichotomum*
*J. odoratissimum*: *Jasminum odoratissimum*
*J. fluminense*: *Jasminum fluminense*
*J. fruticans*: *Jasminum fruticans*
*J. tortuosum*: *Jasminum tortuosum*
*J. polyanthum*: *Jasminum polyanthum*
SP: Single-petal
DP: Double-petal
MP: Multi-petal
cp: Chloroplast
cpDNA: Chloroplast genome
SSR: Simple sequence repeats
SNPs: Single nucleotide polymorphisms
RSCU: Relative synonymous codon usage
CDS: Coding sequences
Ka/Ks: Non-synonymous/synonymous substitution ratio
LSC: Large single-copy
SSC: Small single-copy
IRs: Inverted repeats
PCGs: Protein-coding genes
FRs: Forward repeats
RRs: Reverse repeats
CRs: Complement repeats
PRs: Palindromic repeats
PCA: Principal component analysis
CAI: Codon Adaptation Index
