# Supplementary material for: Twelve newly assembled jasmine chloroplast genomes: unveiling genomic diversity, phylogenetic relationships and evolutionary patterns among Oleaceae and Jasminum species
Source: BMC Plant Biol. 2024 Apr 25;24:331. doi: 10.1186/s12870-024-04995-9 (PMC11044428; doi:10.1186/s12870-024-04995-9)
Supplement: Supplementary file 2 — Supplementary Material 2. [file 12870_2024_4995_MOESM2_ESM.docx]

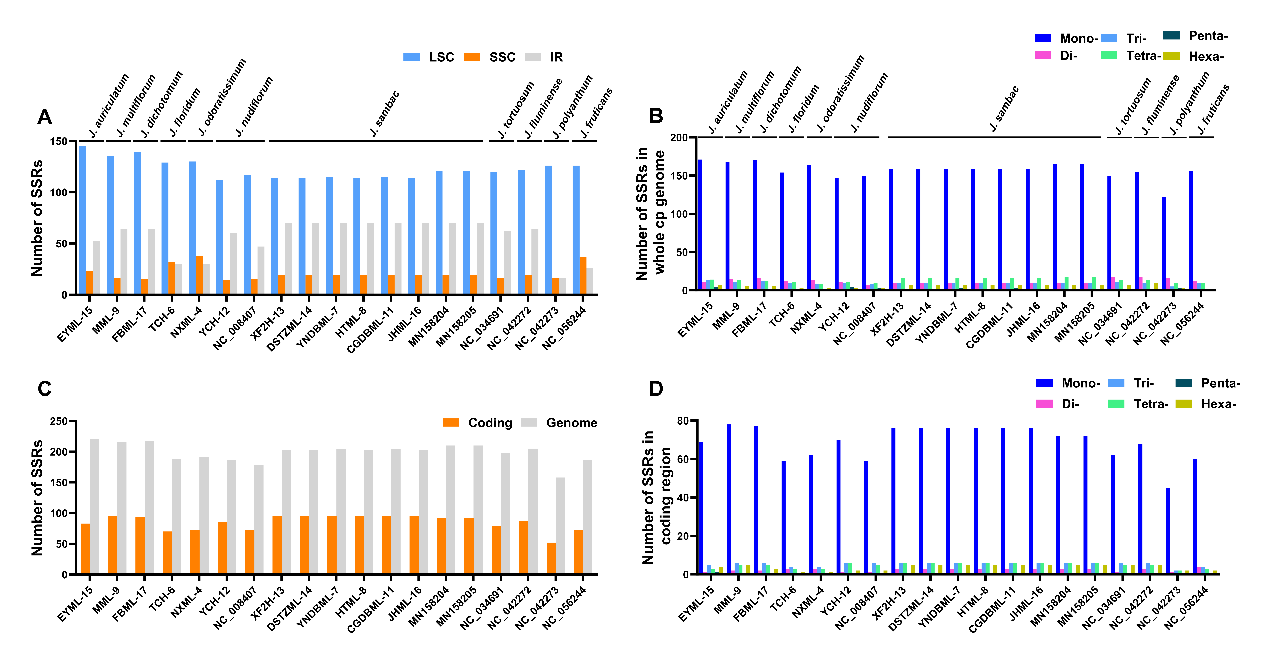


**Fig. S1**. Distribution and frequency of simple sequence repeats (SSRs) in chloroplast genomes of 12 species from *Jasminum*. (A) The total number of SSRs in different regions. (B) The total number of different types SSRs in LSC, SSC, IR regions. (C) The number of SSRs in coding and genomes sequence. (D) The number of different types SSRs in coding sequence.


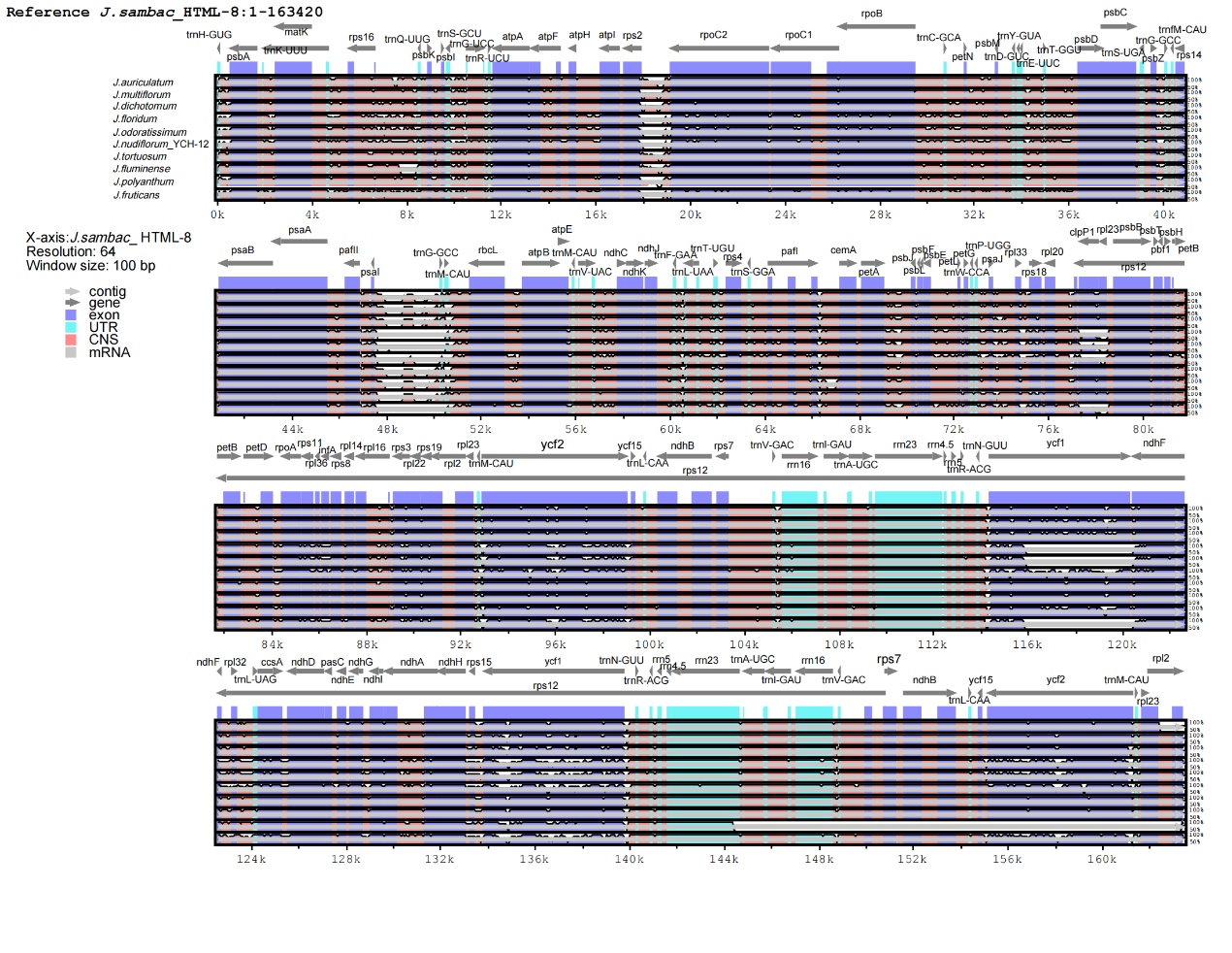


**Fig. S2**. Sequence alignment of chloroplast genomes from 11 species in *Jasminum*. X-axis represents the reference sequence (*Jasminum sambac,* HTML-8), and the y-axis shows the consistency ranges of genomes of each species with reference. Arrow shows the annotated genes with transcriptional directions. Different colors represent the various regions in chloroplast genome.


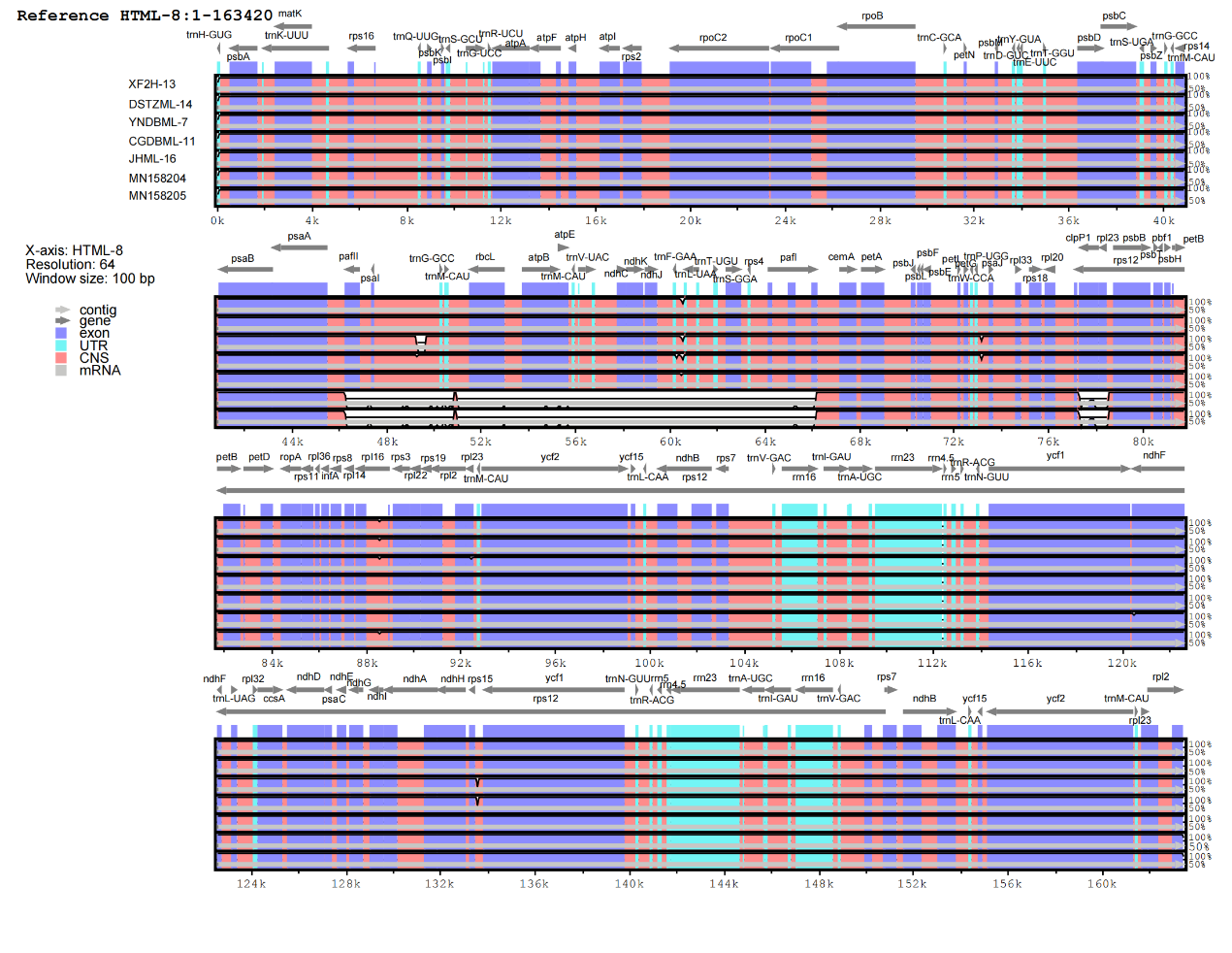


**Fig. S3**. Sequence alignment of chloroplast genomes from 8 samples in *J*. *sambac*. X-axis represents the reference sequence (HTML-8), and the y-axis shows the consistency ranges of genomes of each species with reference. Arrow shows the annotated genes with transcriptional directions. Different colors represent the various regions in chloroplast genome.


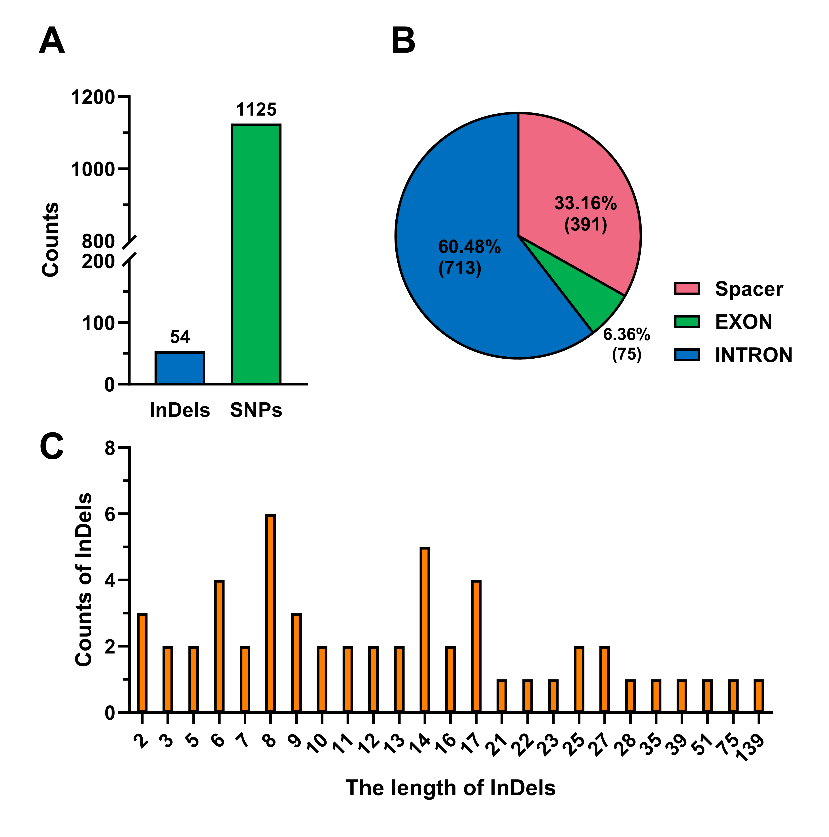


**Fig. S4**. The figure shows the variation in the number (A) and proportion of SNPs and InDels in the spacer, exon, and intron regions, as well as the number of different lengths of InDels (C), in 18 chloroplast genomes from *Jasminum*.


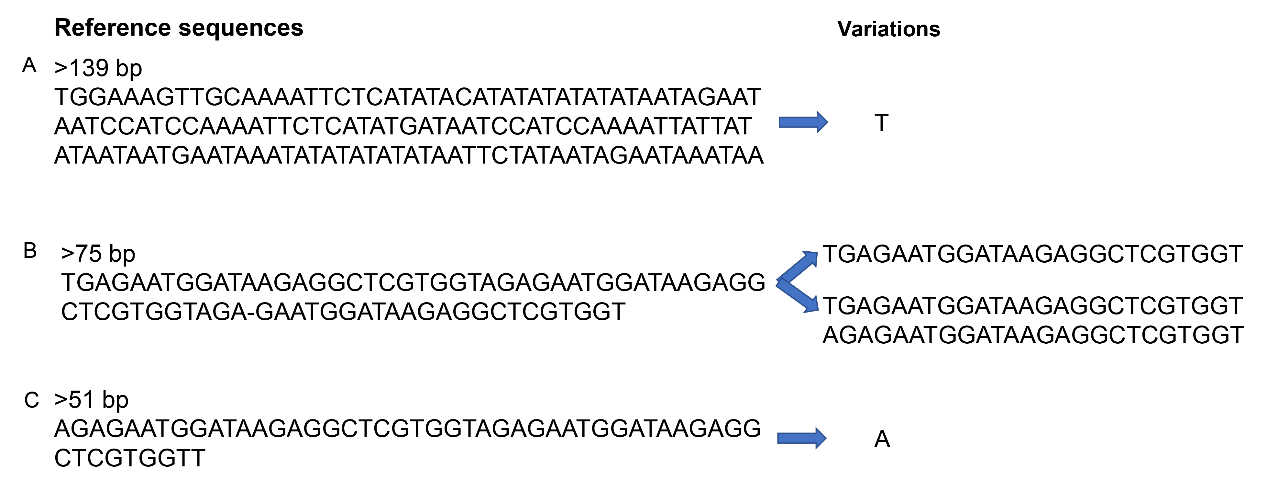


**Fig. S5**. The sequence variation information for the three longest InDels is depicted in A, B, and C. Panel A shows the longest InDel, where a deletion occurs in the sequence from the left 139 bp to T. Panel B represents the second longest InDel, where a sequence of 75 bp mutates into two corresponding scenarios indicated by the arrows. Panel C illustrates the third longest InDel, where a deletion mutation occurs in a sequence of 51 bp from the left to A.
